# Cross-organ multi-omics profiling of microbiome and metabolome along the gut–liver axis in MASH model mice induced by western diet and MC4R knockout

**DOI:** 10.1186/s13099-026-00813-9

**Published:** 2026-02-25

**Authors:** Mitsuharu Matsumoto, Osamu Miura, Takeo Moriya, Hitomi Ogino, Megumi Hirayama, Akira Mitsui, Takaharu Hirayama, Yukihiko Ebisuno, Manami Kaneko, Yoshinori Satomi, Yasunori Nio

**Affiliations:** 1 Pharmacology Business Unit, Axcelead Drug Discovery Partners, Inc, 26-1, Muraoka-Higashi 2-Chome, 251-0012 Fujisawa, Japan; 2 DMPK & Safety Business Unit, Axcelead Drug Discovery Partners, Inc, 26-1, Muraoka-Higashi 2-Chome, 251-0012 Fujisawa, Japan; 3 Project Management, Business Planning & Operations, Axcelead Drug Discovery Partners, Inc, 26-1, Muraoka-Higashi 2-Chome, 251-0012 Fujisawa, Japan

**Keywords:** Metabolic dysfunction-associated steatohepatitis (MASH), Microbiome–metabolome interactions, Intestinal dysbiosis, Gut–liver axis

## Abstract

**Background:**

Western diet (WD) fed Melanocortin 4 receptor-knockout (MC4R-KO) mice develop a phenotype resembling human metabolic dysfunction-associated steatohepatitis (MASH). Despite its clinical relevance, the role of the gut–liver axis in MASH pathogenesis remains unclear. We investigated the gut-liver axis through microbiomic and metabolomic analyses of WD-fed MC4R-KO mice, and we examined their association with MASH pathology.

**Methods:**

We performed an integrated microbiome and metabolome analysis of the liver, small intestinal contents, large intestinal contents, and plasma of wild-type (WT) and MC4R-KO mice fed either a normal diet or WD. Markers of hepatic inflammation, fibrosis, and steatosis measured in this study were used to assess MASH severity and to correlate microbiome and metabolite alterations.

**Results:**

WD-fed MC4R-KO mice exhibited significant hepatic steatosis, inflammation, and fibrosis. The abundance of certain microbiota, including Muribaculaceae and *Allobaculum*, negatively correlated with MASH severity, whereas increased levels of Desulfovibrionaceae and *Bacteroides* positively correlated with hepatic lipid accumulation, steatosis, and inflammation. Metabolomic profiling revealed increased triglyceride and diglyceride levels in the liver and a concomitant decrease in free fatty acids and monoglycerides in the intestines. Additionally, plasma taurine-conjugated bile acids were elevated in WD-fed MC4R-KO mice, which correlated with the reduced hepatic transport of bile salts from pathway enrichment analysis. These findings highlight substantial alterations in the gut microbiota and lipid and bile acid metabolism, indicating a mechanistic dysregulation of the gut–liver axis that may contribute to MASH progression.

**Conclusion:**

The observed gut microbial and metabolic alterations, particularly bile acid and lipid metabolism dysregulation, offer insights into potential therapeutic targets aimed at modulating the gut–liver axis to treat or prevent MASH.

**Supplementary Information:**

The online version contains supplementary material available at 10.1186/s13099-026-00813-9.

## Background

Metabolic dysfunction-associated steatohepatitis (MASH; formerly known as non-alcoholic steatohepatitis (NASH)), a progressive form of metabolic dysfunction-associated steatotic liver disease (MASLD), is the leading cause of chronic liver disease worldwide and is projected to become the most common indication for liver transplantation within the next decade [1]. Its prevalence has shown an upward trend owing to the increasing global burden of obesity, metabolic syndrome, and type 2 diabetes mellitus (T2DM) [2]. To this end, the FDA approved resmetirom (Rezdiffra) in 2024 as the first pharmacological treatment for MASH. However, its pathogenesis is complex and unclear, requiring further elucidation for the development of more efficacious therapeutics. Recent evidence highlighted the gut-liver axis as a pivotal regulator of hepatic homeostasis and a central player in the pathophysiology of MASH [3]. Dysbiosis has been implicated in the disruption of gut barrier integrity, leading to enhanced intestinal permeability ("leaky gut"), translocation of bacterial products, into the portal circulation, and subsequent hepatic inflammation [4]. This endotoxemia-driven inflammatory cascade is considered a major contributor to MASH pathogenesis.

Microbial-derived metabolites, including short-chain fatty acids (SCFAs), bile acids, branched-chain amino acids, and tryptophan catabolites such as indole derivatives, interact with host signaling pathways to modulate lipid metabolism, inflammatory activation, and fibrosis [5]; alterations in microbial metabolic output may serve as a critical functional link bridging gut dysbiosis and hepatic injury in MASH. Recent findings from human cohort studies have clarified the presence of distinct gut microbiome signatures associated with MASH severity, including an increased abundance of pro-inflammatory taxa, such as Enterobacteriaceae, and decreased levels of protective genera, such as *Faecalibacterium* [6]. However, establishing causal relationships remains challenging in humans owing to confounding factors such as diet, medication use, and comorbidities. Consequently, translationally relevant animal models that recapitulate both metabolic and histopathological features of human MASH are essential for mechanistic investigations. Although traditional rodent models of MASH, such as those from methionine- and choline-deficient (MCD) or choline-deficient, L-amino acid-defined (CDAA) diets, reliably induce steatohepatitis and fibrosis, they fail to replicate key clinical hallmarks such as obesity, insulin resistance, and dyslipidemia [7]. Conversely, the melanocortin 4 receptor–deficient (MC4R-KO) mice, when challenged with a Western diet (WD) rich in fat, cholesterol, and sugar, develops obesity, insulin resistance, hyperinsulinemia, dyslipidemia, and progressive liver disease, including advanced steatohepatitis with lobular inflammation, and bridging fibrosis [8, 9]. Furthermore, WD-fed MC4R-KO mice exhibit hepatic transcriptomic profiles and immune cell infiltration patterns similar to those observed in patients with MASH [8]. Despite their increasing use in MASH research, the composition of their gut microbiome and its functional metabolic outputs remain to be systematically characterized. Furthermore, the correlation between microbial community alterations and shifts in metabolite profiles, and their relationship with histological progression of liver disease remains unclear. Herein, we performed an integrated multi-omics characterization of the gut–liver axis in a WD-fed MC4R-KO mouse model to identify the key gut-derived factors that contribute to MASH progression. Through this comprehensive approach, we aimed to generate mechanistic insights into the gut–liver axis in MASH and uncover novel biomarkers and therapeutic avenues for this increasingly prevalent and devastating disease.

## Methods

### Animals and diets

Male C57BL/6 J (WT) mice were purchased from Charles River Laboratories (Yokohama, Japan). Male MC4R KO mice were generated as previously described [45]. Six-week-old MC4R KO mice and WT mice were fed with a standard chow diet (CE-2; CLEA Japan, Tokyo, Japan) for 15 days following feeding with a normal diet (ND, 98,121,701; Research Diets, New Brunswick, Canada) for 12 days in the same room. MC4R KO and WT mice were grouped using stratified random allocation based on plasma insulin, alanine transaminase (ALT), aspartate aminotransferase (AST), tissue inhibitor of metalloproteinase-1 (TIMP-1), triglyceride (TG), total cholesterol, and body weight utilizing the biological experimental data statistical analysis system EXSUS. They were then fed either a WD (D12079B; Research Diets, New Brunswick, Canada) or an ND for 12 weeks from 10 weeks of age. The groups are as follows: WT mice fed with ND (n = 6); WT mice fed with WD (n = 6); MC4R KO mice fed with ND (n = 8); MC4R KO mice fed with WD (n = 8). The mice were allowed ad libitum access to food and water and individually housed under controlled temperature, humidity and a 12-h light–dark cycle (lights on 7:00–19:00). All animal experiments were approved by the Institutional Animal Care and Use Committee of the Shonan Research Center (AU-00021171).

### Tissue sample preparation

Blood was collected from the tail vein before and after 6 and 12 weeks of feeding either ND or WD. The livers were snap-frozen in liquid nitrogen under isoflurane anesthesia (3–5%) after 12 weeks of feeding. The portion from the gastric pylorus to the cecum was divided in half at the midpoint, and the digestive tract contents of a 4 cm portion from the midpoint to the periphery were collected as small intestinal contents. Similarly, the portion from the cecum to the anus was divided in half at the midpoint, and the digestive tract contents of a 4 cm portion from the midpoint to the periphery were collected as the large intestinal contents.

### Plasma analysis

Plasma ALT, AST, TG, and total cholesterol levels were measured enzymatically using Clinical Analyzer 7180 (Hitachi High-Technologies, Tokyo, Japan). Plasma insulin concentrations were measured using an ultrasensitive mouse insulin enzyme-linked immunosorbent assay kit (Morinaga Institute of Biological Science, Kanagawa, Japan). Plasma TIMP-1 concentrations were measured using the Mouse TIMP-1 Quantikine ELISA Kit (R&D Systems, Minneapolis, MN, US). Plasma lipopolysaccharide (LPS) concentrations were measured using Limulus Color KY Test Wako (FUJIFILM Wako Pure Chemical Corporation, Osaka, Japan) according to manufacturer’s protocol after dilution and heat inactivation for 10 min at 70℃.

### Liver analysis

For the measurement of hepatic triglycerides, liver aliquots were homogenized at a concentration of 100 mg of tissue per 1 mL of saline. The homogenate was then thoroughly mixed with hexane and 2-propanol (3:2). After centrifugation, lipid-containing upper organic layers were collected. The collected layers were dried, and the residue was dissolved in 2-propanol. The triglyceride concentration in 2-propanol was measured using the Triglyceride-E test (Fujifilm Wako Pure Chemical Industries, Osaka, Japan). Hepatic hydroxyproline content was measured using a commercially available Sensitive Tissue Collagen Assay Kit (Quickzyme Biosciences, Leiden, Netherlands), according to the manufacturer’s instructions. Total RNA was isolated from 50–100 mg of liver tissue using the RNeasy Mini kit (Qiagen, Tokyo, Japan), followed by reverse transcription using a high-capacity RNA-to-cDNA kit (Thermo Fisher Scientific, Tokyo, Japan) according to the manufacturer’s instructions. The resulting cDNA (2 μL) was subjected to quantitative real-time PCR using 5 μL of TaqMan Fast Advanced Master Mix and 0.5 μL of TaqMan gene expression assay (Applied Biosystems, Tokyo, Japan) in a total volume of 10 μL per reaction on an ABI7900 system (Life Technologies, Tokyo, Japan). Thermocycling conditions consisted of an initial denaturation of 20 s at 95 °C, followed by 40 cycles of 95 °C for 1 s and 60 °C for 20 s. Commercially available primer–probe sets were used (Applied Biosystems, Tokyo, Japan). Those sets were as follows: mouse collagen type1 alpha1 (Col1a1) (Mm00801666), mouse Acta2 (αSMA) (Mm00725412), F4/80 (Mm00802529), transforming growth factor-β1 (TGF-β1) (Mm01159846), tumor necrosis factor alpha (TNF-α) (Mm00443258), MCP1 (Ccl2) (Mm00441242). GAPDH (Mm99999915) served as an endogenous control gene. Relative mRNA expression was calculated by ΔΔCt method.

### Metabolomics

Metabolite profiles were acquired using hydrophilic interaction liquid chromatography/tandem mass spectrometry (HILIC/MS/MS), gas chromatography/tandem mass spectrometry (GC/MS/MS) analyses, and nontargeted lipidomic analyses.

### Metabolite extraction

Plasma metabolites were extracted by adding nine volumes of ice-cold methanol, followed by vortexing. For liver, small and large intestinal contents, frozen tissues were first pulverized using a ShakeMaster Auto (Bio Medical Science Inc., Tokyo, Japan) at 1100 rpm for 1 min. Subsequent to pulverization, metabolites were extracted on ice by adding nine volumes of methanol to the liver and 99 volumes to the small and large intestinal contents. All mixtures were homogenized thoroughly. The resulting extracts were stored at − 80 °C until further analysis.

### HILIC/MS/MS analysis

The extracted samples were mixed with 400 mM ammonium formate at a ratio of 19:1 (sample: ammonium formate, v/v) and centrifuged at 21,500 × g for 5 min at 4 ℃. The final supernatant was analyzed by an LC/MS/MS using a UHPLC Nexera system (Shimadzu Co., Kyoto, Japan) and coupled with a 5500QTRAP mass spectrometer (AB Sciex LLC, MA, USA). Separation was performed on a ZIC-cHILIC column (2.1 × 100 mm, 3 μm, Merck Millipore) maintained 30 °C with a gradient elution of 10 mM ammonium formate aqueous solution (A) and acetonitrile (B), delivered at a flow rate of 0.4 mL/min with an injection volume of 2 μL. The gradient program was optimized as follows: 0–1.5 min, 97% B; 1.5–5 min, 97–75% B; 5–7 min, 75% B; 7–10 min, 75–40% B; 10–12 min, 40% B; 12–13 min, 40–10% B; 13–16 min, 10% B; 16–25 min, 97% B. Mass spectrometry was conducted using electrospray ionization in multiple reaction monitoring (MRM) mode, following previously described parameters [10]. Data were processed using MultiQuant 3.0 software (AB Sciex). Individual metabolites were identified by comparing retention times and MRM transitions with those of authentic standard compounds.

### GC/MS/MS analysis

Stable isotope-labeled internal standards were added to the extracted samples to normalize analytical variations, and the mixtures were subsequently evaporated to dryness under a stream of nitrogen gas.

The dried residues were derivatized by oximation with methoxyamine hydrochloride in pyridine, followed by trimethylsilylation using MSTFA. The derivatized metabolites were analyzed using an Agilent 7890B gas chromatography system coupled with an Agilent 7010 series triple-quadrupole mass spectrometer (Agilent Technologies Inc., Santa Clara, CA, USA). Chromatographic separation was achieved on a DB-5MS column (30 m × 0.25 mm, df = 0.25 μm film thickness; Agilent Technologies Inc.) using helium as the carrier gas at a consistent flow rate of 1 mL/min. The oven temperature was controlled by a programmed gradient. The eluent was ionized via electron impact and analyzed using MRM mode. Data acquisition and processing were performed using MassHunter software (Agilent Technologies Inc.).

### Non-targeted lipidomic analysis

Lipidomic analyses were performed as described previously [11]. Lipids were extracted using ethanol containing 0.002% butylated hydroxytoluene to prevent oxidation, for plasma and intestinal contents (small and large), samples were mixed with an equal volume of the extraction solvent. For liver samples, four volumes of the solvent were added. After vigorous vortexing, the mixtures were centrifuged at 21,500 × g for 5 min at 4 °C. The resulting supernatant (2μL) was injected onto a CORTECS T3 column (2.1 × 100 mm, 2.7 μm; Waters, MA, USA) maintained at 40 °C. Chromatographic separation was performed by gradient elution using an aqueous mobile phase (A; MilliQ water with 0.01% acetic acid, 1 mM NH_3_, and 10 μM EDTA-2Na) and an organic mobile phase (B; ethanol/isopropanol [1:1] containing 0.001% acetic acid and 0.2 mM NH_3_), at a flow rate of 0.7 mL/min. The gradient program was as follows: 0–1 min, 0% B; 1–13 min, 0–100% B; 13–15 min, 100% B; 15–18 min, 0% B. Eluents were introduced into a Q Exactive HF-X mass spectrometer (Thermo Fisher Scientific, Waltham, MA, USA). Mass spectra were acquired in the data-dependent acquisition mode. Precursor and product ion spectra were generated by higher energy collisional dissociation and analyzed using an Orbitrap analyzer at resolutions of 120,000 and 7,500 full width at half maximum at 200 m/z, respectively. Raw LC/MS data were processed using Expressionist Refiner MS software (ver. 8.2; GeneData AG, Basel, Switzerland). Each MS peak was assigned by comparison with an in-house lipid database, containing information on retention time, exact mass, preferred adduct ion species, and chemical structure.

### Internal standards and data correction

In the GC/MS/MS analysis, isotope-labeled compounds were added to each sample as internal standards to correct for variability introduced during derivatization and instrumental analysis. The detected metabolite signals were normalized to these internal standards.

For HILIC/MS/MS analysis and non-targeted lipidomic analysis, external standards were analyzed to verify retention times, monitor instrument performance, and ensure accurate peak identification.

### Quality control

To monitor analytical reproducibility across all metabolomic platforms, a pooled quality control sample (mixQC) was prepared by combining aliquots from representative biological samples. The mixQC was analyzed after every five to six study samples throughout the measurement sequence. The coefficient of variation (CV) of detected metabolites in the mixQC was maintained below 30%, ensuring acceptable analytical precision and instrument stability.

### 16S rRNA amplicon sequencing

Approximately 100–200 mg of small or large intestinal contents from WD-fed or ND-fed MC4RKO and WT mice were freeze-dried using a VD-250R Freeze Dryer (TAITEC). The freeze-dried samples were homogenized at 1,500 rpm for 2 min using a multi-bead shocker (Yasui Kikai, Osaka, Japan). The DNA was extracted as follows: Lysis Solution F (Nippon Gene, Tokyo, Japan) was added to the crushed samples and incubated at 65 °C for 10 min. The samples were then centrifuged at 12,000 × g for 2 min, and the supernatant was collected. DNA was purified from the collected solution using the MPure-12 system and MPure Bacterial DNA Extraction Kit (MP Biomedicals, Tokyo, Japan). The concentration of the purified DNA was measured using Synergy LX (BioTek, Winooski, Vermont, USA) and QuantiFluor dsDNA System (Promega, Madison, WI, USA). The sequencing library was prepared using a 2-step tailed PCR method. In the first PCR, 16S region-specific primers were used to amplify the V3-V4 region:

341f_MIX:5’-ACACTCTTTCCCTACACGACGCTCTTCCGATCTNNNNNCCTACGGGNGGCWGCAG-3’.

805r_MIX:5’-GTGACTGGAGTTCAGACGTGTGCTCTTCCGATCTNNNNNGACTACHVGGGTATCTAATCC-3’.

Each 10 μL reaction contained 0.05 U/μL of RxTaq HS (Takara Bio, Shiga, Japan), 0.5 μM of each primer, and 1 ng of template DNA. The PCR conditions were as follows: initial denaturation at 94 °C for 2 min; 30 cycles of 94 °C for 30 s, 55 °C for 30 s, and 72 °C for 30 s; followed by a final extension at 72 °C for 5 min. PCR products were cleaned using AMPure XP beads (Beckman Coulter, CA, USA) following the manufacturer’s protocol. A second PCR was performed using the following indexed primers:

F:5’-AATGATACGGCGACCACCGAGATCTACAC-Index2 ACACTCTTTCCCTACACGACGC-3’.

R:5’-CAAGCAGAAGACGGCATACGAGAT-Index1 GTGACTGGAGTTCAGACGTGTG-3’.

In this step, cleaned amplicons from the first PCR served as templates in 10 μL reactions with 0.05 U/μL of RxTaq HS (Takara Bio) and 0.5 μM of each indexed primer. Amplification was conducted for 10 cycles under the same cycling conditions as the first PCR. The concentration of the library was measured using Synergy H1 (BioTek) and QuantiFluor dsDNA System. Library quality was assessed using a Fragment Analyzer and a dsDNA 915 Reagent Kit (Advanced Analytical Technologies, Orangeburg, NY, USA). DNA sequencing was performed on the MiSeq system using the MiSeq Reagent Kit v3 (Illumina, San Diego, CA, USA), generating 2 × 300 bp paired-end reads.

### Data analysis

Read sequences (39,000–60,000 paired-end reads) whose starting sequences perfectly matched the primer sequences used were extracted using the fastx_barcode_splitter tool from FASTX-Toolkit (ver. 0.0.14). When the primer sequences contained N-mix, this process was repeated, considering the number of Ns (six types on the forward side × six types on the reverse side = 36 types). The extracted reads were trimmed to remove primer sequences using fastx_trimmer from the FASTX-Toolkit. Sequences with a quality score < 20 were removed using Sickle (ver. 1.33), and sequences shorter than 130 bases along with their paired sequences were discarded. Paired-end read merging was performed using FLASH script (ver. 1.2.11). After removing chimeric and noisy sequences using the DADA2 plugin in Qiime2 (ver. 2021.4), 23,000–35,000 amplicon sequence variants (ASVs) and their feature table were generated. Taxonomic classification was performed by assigning the ASV sequences against Greengenes database (ver. 13_8) using a feature classifier plugin. Alpha and beta diversity analyses were performed using the diversity plugin in QIIME 2. Group comparisons of alpha diversity were based on the Shannon index, taxa richness, and Pielou’s evenness, and the differences between groups were assessed using the Kruskal–Wallis test. Group comparisons of beta diversity were conducted using Jaccard distance, Bray–Curtis distance, unweighted UniFrac distance, and weighted UniFrac distance, and differences between groups were assessed using PERMANOVA (Permutational Multivariate Analysis of Variance).

### Statistics

Data for MASH phenotypic markers including plasma, liver parameters and gene expression are represented as mean ± S.D. For the evaluation of the influences of genetic background and diet type differences, statistical differences were analyzed using Tukey-test or Steel–Dwass test. Statistical significance was set at P < 0.05. For the interaction effects of diet and genetic background, a two-way analysis of variance (ANOVA) was performed and statistical significance was set at P < 0.05.

Group comparisons of hepatic metabolites were conducted using the Wilcoxon rank-sum test, with P-values adjusted using the Benjamini–Hochberg false discovery rate (FDR) correction for multiple-testing. Metabolites exhibiting large variations were defined as those with |log_2_ fold change|≥ 2.5 and adjusted P-value < 0.05. Pathway enrichment analysis for significantly altered hepatic metabolites (adjusted P-value < 0.05) was performed using the Ingenuity Pathway Analysis (IPA) (Fall 2024 release). Group comparisons in the microbiome analysis were conducted using LEfSe. The taxa exerting the greatest influence on the separation of the groups were defined as the top ten genera with the highest absolute LDA scores. The abundances of bacterial enzymes and their pathways were predicted using PICRUSt2. It should be noted that these predictions are inferred from 16S rRNA-based microbiome data and represent putative functional potential rather than direct measures of enzymatic activity. Group comparisons of predicted enzyme and pathway abundances were conducted in the same manner as described above for hepatic metabolites using the Wilcoxon rank-sum test, with P-values adjusted using the Benjamini–Hochberg method.

### Weighted gene co-expression network analysis (WGCNA)

WGCNA, a clustering technique that leverages pairwise correlation coefficients (correlation matrices), was used to classify the microbiota, enzymes inferred from PICRUSt2, and metabolites based on their signal intensities, using R (version 4.3.3) and the ‘WGCNA’ package (version 1.72–5). Initially, the signal intensities of the individual microbiota and molecules from all samples (n = 28) were transformed into robust Z-scores for normalization, and the resulting robust Z-scores were used as the input matrix for WGCNA. To construct a weighted co-expression network, the soft-thresholding power value (β) was set at 4, as this was the minimum value at which the scale-free topology fit index curve stabilized. To minimize noise and spurious correlations, the adjacency matrices, constructed using Spearman’s rank-based correlation coefficients, were converted into a topological overlap matrix (TOM), with the corresponding dissimilarities (dissTOM) computed as 1 – TOM. For hierarchical clustering, dissTOM was used as the distance metric. The minimum module size was set to 30 with a merge cut height of 0.2. Overall, microbiota, enzymes, and metabolites were classified into 31 modules. To identify modules strongly associated with the phenotypic markers of MASH, correlation analyses were conducted between each module and its signal intensities. These relationships were assessed using Spearman’s correlation coefficients between module eigengenes (MEs), which represent the first principal component of signal intensities within a module, and phenotypic markers of MASH. Modules that exhibited significant correlations with disease severity in WD-fed and/or MC4R KO mice were selected for further investigation. Specifically, the interactions between markedly altered hepatic metabolites and other key microbiota and metabolites, including those present in the small and large intestinal contents, were analyzed based on WGCNA results.

## Results

### Phenotypic characterization of MASH Model Mice

To determine the influence of genotype and dietary intervention on MASH pathogenesis, four experimental groups were established: normal diet-fed wild-type (WT-ND), western diet-fed wild-type (WT-WD), normal diet-fed MC4R-KO (KO-ND), and western diet-fed MC4R-KO (KO-WD) mice. After 12 weeks of feeding, phenotypic, biochemical, and gene expression analyses were performed for all groups (Tables 1–2). Only male mice were used to minimize the effects of estrogen-mediated hepatoprotection on lipid metabolism, inflammation, and fibrosis [12–15].

**Table 1:** Phenotypic profiles of plasma, liver, and body parameters

Parameters	Unit	WT-ND	WT-WD	KO-ND	KO-WD
Body weight	g	33.8 ± 1.8	43.4 ± 1.1^a^	49.3 ± 2.0^ab^	59.9 ± 4.1^abc^
Liver weight	g	1.41 ± 0.14	2.71 ± 0.30^a^	3.29 ± 0.67^a^	7.32 ± 1.10^abc^
ALT	U/L	25.7 ± 7.1	205.8 ± 91.2^a^	341.9 ± 170.1^a^	774.3 ± 104.3^abc^
AST	U/L	67.5 ± 11.9	187.4 ± 91.4^a^	384.2 ± 187.1^a^	605.8 ± 90.9^ab^
Plasma insulin	ng/mL	1.89 ± 0.71	3.33 ± 0.70	8.77 ± 3.04^ab^	26.29 ± 9.64^abc^
Plasma TIMP-1	ng/mL	1.54 ± 0.20	2.54 ± 0.58^a^	3.25 ± 0.80^a^	8.39 ± 1.47^abc^
Plasma triglyceride	mg/dL	65.2 ± 17.9	50.6 ± 12.4	32.7 ± 10.1^a^	83.0 ± 16.7^bc^
Plasma total cholesterol	mg/dL	173.3 ± 23.1	291.8 ± 36.0^a^	207.4 ± 45.2^b^	377.4 ± 25.7^abc^
Plasma LPS^*)^	EU/mL	1.70 ± 2.28	3.24 ± 4.70	1.49 ± 3.51	2.91 ± 3.28
Hepatic hydroxyproline content	μg/mg tissue	4.69 ± 0.76	4.57 ± 0.68	6.33 ± 0.95^ab^	9.40 ± 1.36^abc^
Hepatic triglyceride content	mg/g tissue	23.3 ± 11.7	79.4 ± 21.0^a^	63.4 ± 13.6^a^	65.7 ± 9.2^a^

MC4R knockout (KO) mice and wild-type (WT) mice were fed either a Western diet (WD) or a normal diet (ND) for 12 weeks. Values were obtained after 12 weeks of feeding with a WD or ND. The groups were as follows: WT-ND, WT mice fed a ND (n = 6); WT-WD, WT mice fed a WD (n = 6); KO-ND, MC4R KO mice fed a ND (n = 8); and KO-WD, MC4R KO mice fed a WD (n = 8). ^*)^ Plasma LPS was measured after 6 weeks of feeding with a WD or ND. Data are presented as mean ± standard deviation (S.D.). Superscript letters indicate statistically significant differences (p < 0.05), as determined by Tukey’s test or Steel–Dwass test: ^a^: P < 0.05 vs. WT-ND,　^b^: P < 0.05 vs. WT-WD, ^c^: P < 0.05 vs. KO-ND

**Table 2:** Summary of statistical analysis by a two-way analysis of variance of plasma, liver, and body parameters

Parameters	Genetic background (WT vs KO)	Diet (ND vs WD)	Interaction
Body weight	P < 0.01, F (1, 24) = 101.46	P < 0.01, F (1, 24) = 246.16	NS, F (1,24) = 0.24
Liver weight	P < 0.01, F (1, 24) = 113.03	P < 0.01, F (1, 24) = 142.95	P < 0.01, F (1,24) = 25.21
ALT	P < 0.01, F (1, 24) = 55.09	P < 0.01, F (1, 24) = 100.44	P < 0.01, F (1,24) = 8.16
AST	P < 0.01, F (1, 24) = 15.42	P < 0.01, F (1, 24) = 64.40	NS, F (1,24) = 1.23
Plasma insulin	P < 0.01, F (1, 24) = 26.38	P < 0.01, F (1, 24) = 50.86	P < 0.01, F (1,24) = 14.77
Plasma TIMP-1	P < 0.01, F (1, 24) = 88.70	P < 0.01, F (1, 24) = 109.10	P < 0.01, F (1,24) = 32.79
Plasma triglyceride	P < 0.01, F (1, 24) = 16.91	NS, F (1, 24) = 0.00	P < 0.01, F (1,24) = 34.44
Plasma total cholesterol	P < 0.01, F (1, 24) = 130.92	P < 0.01, F (1, 24) = 20.95	NS, F (1,24) = 3.89
Plasma LPS^*)^	NS, F (1, 24) = 1.22	NS, F (1, 24) = 0.04	NS, F (1, 24) = 0.00
Hepatic hydroxyproline content	P < 0.01, F (1, 24) = 20.10	P < 0.01, F (1, 24) = 70.86	P < 0.01, F (1,24) = 17.26
Hepatic triglyceride content	P < 0.01, F (1, 24) = 22.57	P < 0.05, F (1, 24) = 5.99	P < 0.01, F (1,24) = 25.06

^*)^ Plasma LPS was measured after 6 weeks of feeding with a WD or ND

Body weight was significantly increased in KO-WD mice compared with WT-ND, WT-WD, and KO-ND mice. Plasma insulin levels were also markedly elevated in the KO-WD group, indicating insulin resistance. Liver injury markers, including alanine aminotransferase (ALT) and AST, were significantly higher in KO-WD mice than in the other three groups. In addition, plasma levels of TIMP-1, a surrogate marker of fibrosis, were significantly increased in KO-WD mice.

The overall severity of metabolic and hepatic phenotypes followed a genotype- and diet-dependent gradient (WT-ND < WT-WD ≤ KO-ND < KO-WD). Two-way ANOVA demonstrated that MC4R deficiency and western diet feeding exerted additive or synergistic effects on multiple phenotypic parameters, with the most pronounced changes observed in KO-WD mice Table 2.

Histological and molecular analyses further supported this gradient. KO-ND mice exhibited hepatic steatosis accompanied by modest increases in hepatic hydroxyproline content and elevated mRNA expression of α-smooth muscle actin and TGF-β1, indicating early fibrotic changes. In contrast, KO-WD mice showed markedly increased hepatic fibrosis together with significant upregulation of fibrosis- and inflammation-associated genes, including Col1a1, TGF-β1, and TNF-α (Table 3).

**Table 3:** Phenotypic expression of mRNA in the liver

Genes	Unit	WT-ND	WT-WD	KO-ND	KO-WD
Col1a1	Fold change	1.00 ± 0.73	7.16 ± 2.39^a^	3.06 ± 1.43^b^	12.38 ± 4.68^ac^
α-SMA		1.00 ± 0.28	1.50 ± 0.39	1.80 ± 0.40^a^	2.11 ± 0.43^ab^
F4/80		1.00 ± 0.40	2.20 ± 0.71	1.88 ± 0.83	3.71 ± 1.73^ac^
TNF-α		1.00 ± 0.45	2.67 ± 0.78^a^	1.84 ± 1.23	3.61 ± 1.27^ac^
MCP-1		1.00 ± 0.48	4.10 ± 1.63^a^	3.46 ± 2.58	9.04 ± 2.95^abc^
TGF-β1		1.00 ± 0.20	1.87 ± 0.28^a^	1.61 ± 0.37^a^	2.47 ± 0.36^abc^

MC4R knockout (KO) mice and wild-type (WT) mice were fed either a Western diet (WD) or a normal diet (ND) for 12 weeks. Values were obtained after 12 weeks of feeding with a WD or ND. The groups are as follows: WT-ND, WT mice fed with ND (n = 6); WT-WD, WT mice fed with WD (n = 6); KO-ND, MC4R KO mice fed with ND (n = 8); KO-WD, MC4R KO mice fed with WD (n = 8). Hepatic mRNA expression levels of Col1a1, α-SMA, F4/80, TNF-α, MCP-1, and TGF-β1 were measured by qPCR and normalized to the WT-ND group. Data are presented as mean ± standard deviation (S.D.). Superscript letters indicate statistically significant differences (p < 0.05), as determined by Tukey’s test or Steel–Dwass test: ^a^: P < 0.05 vs. WT-ND,^b^: P < 0.05 vs. WT-WD, ^c^: P < 0.05 vs. KO-ND

Circulating plasma LPS levels were measured after 6 weeks of dietary intervention. Plasma LPS concentrations tended to increase with western diet feeding, indicating enhanced endotoxemia in WD-fed groups Table 4.

**Table 4:** Summary of statistical analysis by a two-way analysis of variance of mRNA in the liver

Genes	Genetic background (WT vs KO)	Diet (ND vs WD)	Interaction
Col1a1	P < 0.01, F (1, 24) = 53.48	P < 0.01, F (1, 24) = 10.95	NS, F (1,24) = 2.06
α-SMA	P < 0.05, F (1, 24) = 7.17	P < 0.01, F (1, 24) = 23.04	NS, F (1,24) = 0.39
F4/80	P < 0.01, F (1, 24) = 13.99	P < 0.01, F (1, 24) = 8.08	NS, F (1,24) = 0.57
TNF-α	P < 0.01, F (1, 24) = 19.43	P < 0.05, F (1, 24) = 5.04	NS, F (1,24) = 0.02
MCP-1	P < 0.01, F (1, 24) = 28.09	P < 0.01, F (1, 24) = 18.48	NS, F (1,24) = 2.07
TGF-β1	P < 0.01, F (1, 24) = 50.96	P < 0.01, F (1, 24) = 24.60	NS, F (1,24) = 0.00

When compared across commonly used MASH-related phenotypic parameters, WD-fed MC4R KO mice exhibited obesity, insulin resistance, liver injury, steatosis, inflammation, and fibrosis (Table 5).

**Table 5:** Comparative characteristics of MASH model mice used in this study and commonly employed strains

	ALT increase	Steatosis	Fibrosis	Inflammation	Insulin resistance	Obesity
WD-fed MC4R KO mice	〇	〇	〇	〇	〇	〇
ND-fed MC4R KO mice	〇	〇	〇	Δ	ND	〇
CDAHFD-fed WT mice	〇	〇	〇	〇	×	×
CCl4-treated mice	〇	×	〇	〇	ND	×

Summary of key pathological features observed in various mouse models of liver disease. “〇” indicates presence of the corresponding pathological feature, “ × ” indicates absence, “Δ” indicates mild or partial presence, and “ND” indicates not determined

WD-fed MC4R KO mice: MC4R knockout mice fed a Western diet

ND-fed MC4R KO mice: MC4R knockout mice fed a normal diet

CDAHFD-fed WT mice: Wild-type mice fed a choline-deficient, L-amino acid-defined, high-fat diet (CDAHFD). CCl₄-treated mice: Mice administered carbon tetrachloride (CCl₄) to induce liver injury. The pathological features include plasma ALT elevation (ALT increase), hepatic steatosis, fibrosis, inflammation, insulin resistance, and obesity

### Tissue-specific metabolomic alterations

**Fig. 1:**
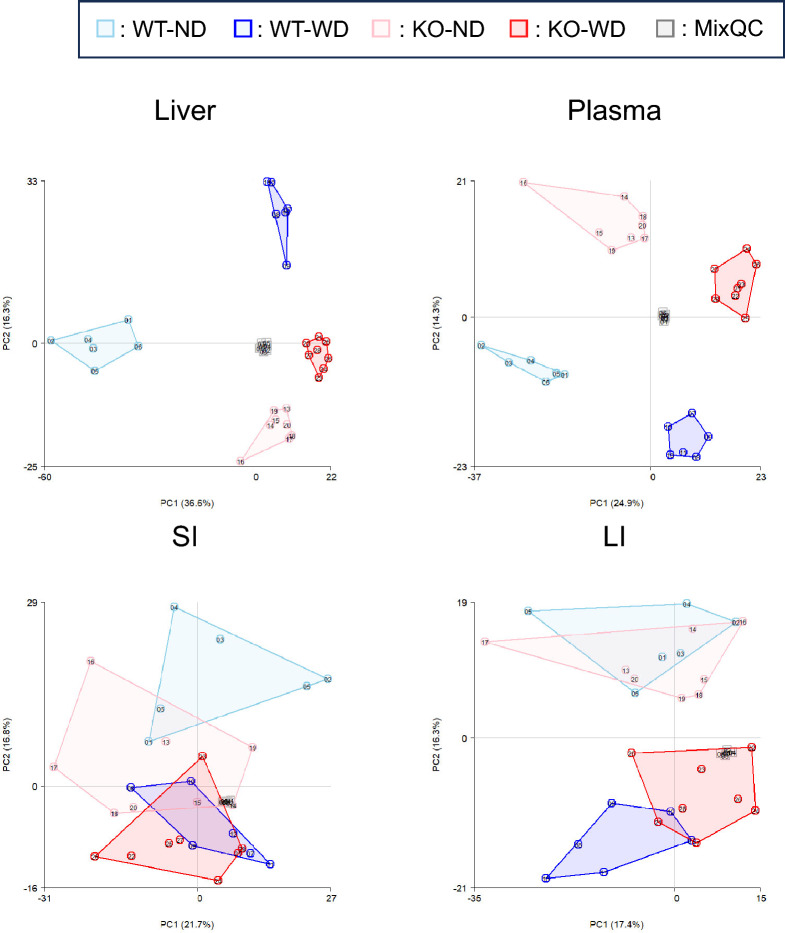
Two-dimensional PCA score plots of metabolomics data from liver, plasma, and intestinal contents. PCA, principal component analysis; WT-ND, normal diet-fed wild-type mice (n = 6); WT-WD, Western diet-fed wild-type mice (n = 6); KO-ND, normal diet-fed MC4R knockout mice (n = 8); KO-WD, Western diet-fed MC4R knockout mice (n = 8); MixQC, pooled quality control (QC) samples prepared by mixing equal aliquots from all experimental samples (n = 7); SI, small intestinal contents; LI, large intestinal contents. PC1 = 36.6% and PC2 = 16.3% for liver; PC1 = 24.9% and PC2 = 14.3% for plasma; PC1 = 21.7% and PC2 = 16.8% for SI; PC1 = 17.4% and PC2 = 16.3% for LI

**Fig. 2:**
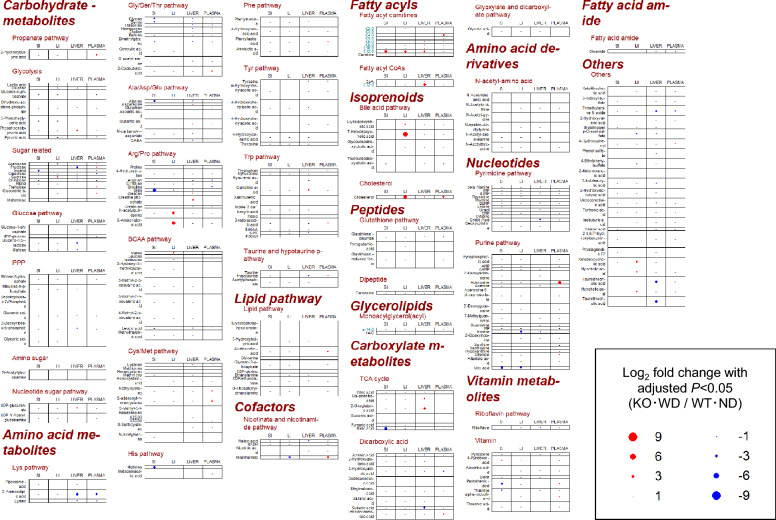
Hydrophilic metabolites across the gut–liver axis and plasma in WD-fed MC4R KO mice. Comprehensive profiling of hydrophilic metabolites showing differences in abundance in the small intestinal contents (SI), large intestinal contents (LI), liver, and plasma obtained from melanocortin-4 receptor knockout (MC4R KO) mice fed a western diet (WD) for 12 weeks (n = 8) and wild-type (WT) mice fed a normal diet (ND) (n = 6). Each colored dot represents a metabolite that exhibits a statistically significant difference in relative abundance between the KO-WD and WT-ND groups. Statistical significance was determined by Wilcoxon rank-sum test, followed by Benjamini–Hochberg false discovery rate (FDR) correction to account for multiple comparisons, with adjusted p < 0.05. Red dots indicate significantly increased metabolites in KO-WD mice, whereas blue dots indicate significantly decreased metabolites. Hyphen (–) denotes metabolites that were not detected in the tissue, intestinal contents, or biofluid. Metabolites are categorized according to their major biochemical pathways, including carbohydrate metabolism (e.g., glycolysis and pentose phosphate pathway), amino acid metabolism (e.g., BCAA, Cys/Met, and Trp), lipid metabolism (e.g., fatty acyl carnitines and bile acids), nucleotides (purine and pyrimidine pathways), cofactors (e.g., nicotinate and riboflavin), and vitamins. Notably, saturated lipid species, such as specific fatty acyl carnitines and bile acid derivatives, are highlighted in blue-green, allowing the visual identification of lipid absorption, hepatic transformation, and systemic circulation in the context of MC4R deficiency and high-fat dietary stress. This figure provides a comprehensive tissue-specific and systemic snapshot of the metabolic perturbations along the gut–liver axis associated with the development of steatohepatitis and metabolic dysfunction in KO-WD mice

**Fig. 3:**
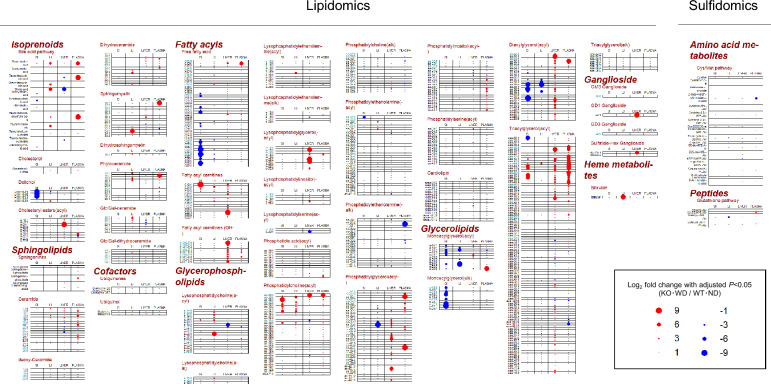
Lipidomic/sulfidomic metabolites across the gut–liver axis and plasma in WD-fed MC4R KO mice. Comprehensive profiling of lipidomic and sulfidomic metabolites (sulfidomic metabolites, i.e., sulfur respiration–related metabolites) showing differences in abundance in the SI, LI, liver, and plasma from MC4R KO mice fed WD for 12 weeks (n = 8), and WT mice fed ND (n = 6). Each dot represents a lipid- or sulfur-containing metabolite exhibiting a statistically significant difference in signal intensity between the KO-WD and WT-ND groups. Statistical significance was determined using Wilcoxon rank-sum test followed by Benjamini–Hochberg correction for multiple testing (FDR-adjusted p < 0.05). Red and blue dots indicate the metabolites that were significantly increased or decreased, respectively, in the KO-WD group. A hyphen (–) indicates that the corresponding metabolite was not detected in the tissue or biofluid. Metabolites were classified into major lipid categories, including fatty acids, glycerophospholipids, glycerolipids, sphingolipids, isoprenoids (e.g., cholesterol and bile acid precursors), gangliosides, heme metabolism–related metabolites (e.g., bilirubin), and lipid cofactors (e.g., ubiquinone). Sulfur-containing gangliosides and sulfolipids are also present. The size of each dot reflects the magnitude of the fold change, and the color indicates directionality. Lipids highlighted in blue-green represent saturated lipid species, which are of particular relevance in metabolic syndrome and steatohepatitis**.** This comprehensive multi-tissue lipidomic and sulfidomic dataset highlights tissue-specific dysregulation of lipid metabolism in the KO-WD model, including enhanced accumulation of ceramides, lysophosphatidylcholines, and triglycerides along the gut–liver axis, providing mechanistic insights into lipid-driven hepatic and intestinal pathophysiology

To investigate the metabolic derangements associated with MASH progression in our model, untargeted metabolomic profiling of liver, plasma, and intestinal contents was performed using high-resolution mass spectrometry. Principal component analysis (PCA) of the metabolomic data revealed a clear separation between the metabolic profiles of the KO-WD mice and those of the other three groups, indicating profound tissue-specific metabolic remodeling (Fig. 1). Subsequently, to capture individual molecular alterations, we generated differential metabolite profiles, focusing on the most contrasting comparison, WT-ND versus KO-WD (Fig. 2 and 3). In hepatic tissues, KO-WD mice exhibited a marked accumulation of triglyceride species, particularly long-chain and polyunsaturated triglycerides, consistent with steatosis. Quantitative analysis revealed that the total hepatic triglyceride content was approximately 2.8-fold higher than that in WT-ND mice (Table 1). Furthermore, distinct alterations in lipid subclasses were observed; diacylglycerols (DAGs) and ceramides, known mediators of lipotoxicity and insulin resistance, were significantly elevated (e.g., Cer(18:1) was significantly increased by more than fourfold [adjusted P < 0.05], and DG (aa-30:1) was significantly increased by more than 175-fold). The reported fold changes represent relative MS signal differences and, especially for low-abundance species or features close to the detection limit, may reflect true biological differences. However, they should be interpreted as approximate estimates, which could be biased upward or downward. Collectively, these metabolomic shifts indicate marked alterations in hepatic and intestinal metabolite profiles in KO-WD mice.

### Plasma metabolomic alterations

Untargeted metabolomic analysis of plasma samples revealed distinct metabolic differences among the experimental groups (Fig. 1). Principal component analysis demonstrated a clear separation of KO-WD mice from WT-ND mice. Differential metabolite analysis comparing KO-WD and WT-ND mice identified a substantial number of significantly altered plasma metabolites (adjusted *P* < 0.05), as summarized in the dot plots (Figs. 2 and 3). These changes predominantly involved lipid- and energy-related metabolites. In particular, multiple lipid-associated species, including triglyceride- and diacylglycerol-related metabolites, as well as bile acid–related metabolites, were significantly increased in the plasma of KO-WD mice.

Consistent patterns were observed when viewed from a pathway perspective, in which plasma metabolites exhibited shifts overlapping with metabolite changes mapped to lipid and bile acid metabolism pathways detected in hepatic and intestinal compartments (Fig. 3). These results demonstrate that WD-fed MC4R KO mice display marked alterations in circulating metabolite profiles, reflecting systemic metabolic changes accompanying the MASH phenotype.

### Shared metabolic signatures across intestinal, hepatic, and plasma compartments

To directly evaluate metabolic features shared across tissues, we compared significantly altered metabolites among the intestinal, liver, and plasma compartments. When viewed from a pathway perspective, overlapping alterations in lipid- and bile acid–related pathways were observed across the small intestinal contents (SI), liver, and large intestinal contents (LI) (Fig. 3).

At the metabolite level, coordinated changes were observed across compartments. Triglyceride- and diacylglycerol-related metabolites were markedly increased in the liver and plasma of KO-WD mice, whereas corresponding reductions in free fatty acids and monoacylglycerols were detected in intestinal contents, particularly in the SI (Fig. 3). In addition, several bile acid species showed concurrent increases in both hepatic and plasma compartments (Fig. 3).

Network-based analyses further supported these cross-compartment associations. Weighted correlation network analysis identified metabolite modules significantly correlated with MASH-related phenotypes, including ALT levels, insulin resistance, and hepatic triglyceride content (Fig. 8). Integration of intestinal metabolites, microbial features, and hepatic metabolites revealed direct associations linking intestinal lipid-related metabolites with hepatic lipid species (Fig. 9). Together, these analyses provide direct evidence of coordinated metabolic alterations across the gut, liver, and circulation in WD-fed MC4R KO mice.

### Intestinal microbiome dysbiosis

**Fig. 4:**
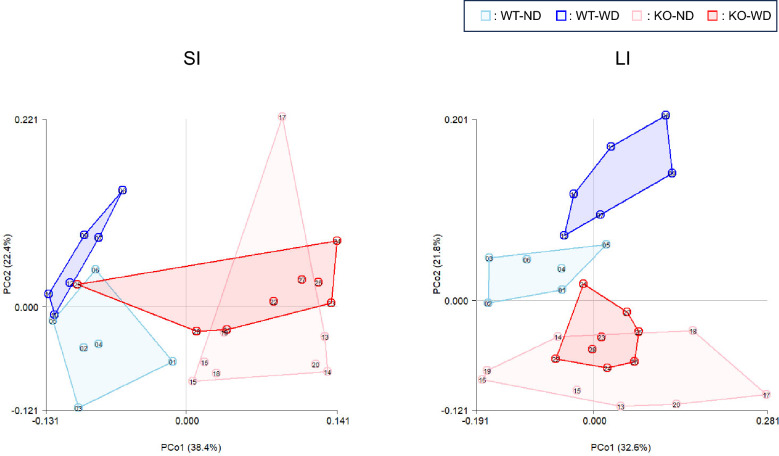
Two-dimensional PCoA score plots of microbiome data from intestinal contents. PCoA, principal coordinate analysis; WT-ND, normal diet-fed wild-type mice (n = 6); WT-WD, Western diet-fed wild-type mice (n = 6); KO-ND, normal diet-fed MC4R knockout mice (n = 8); KO-WD, Western diet-fed MC4R knockout mice (n = 8); SI, small intestinal contents; LI, large intestinal contents

**Fig. 5:**
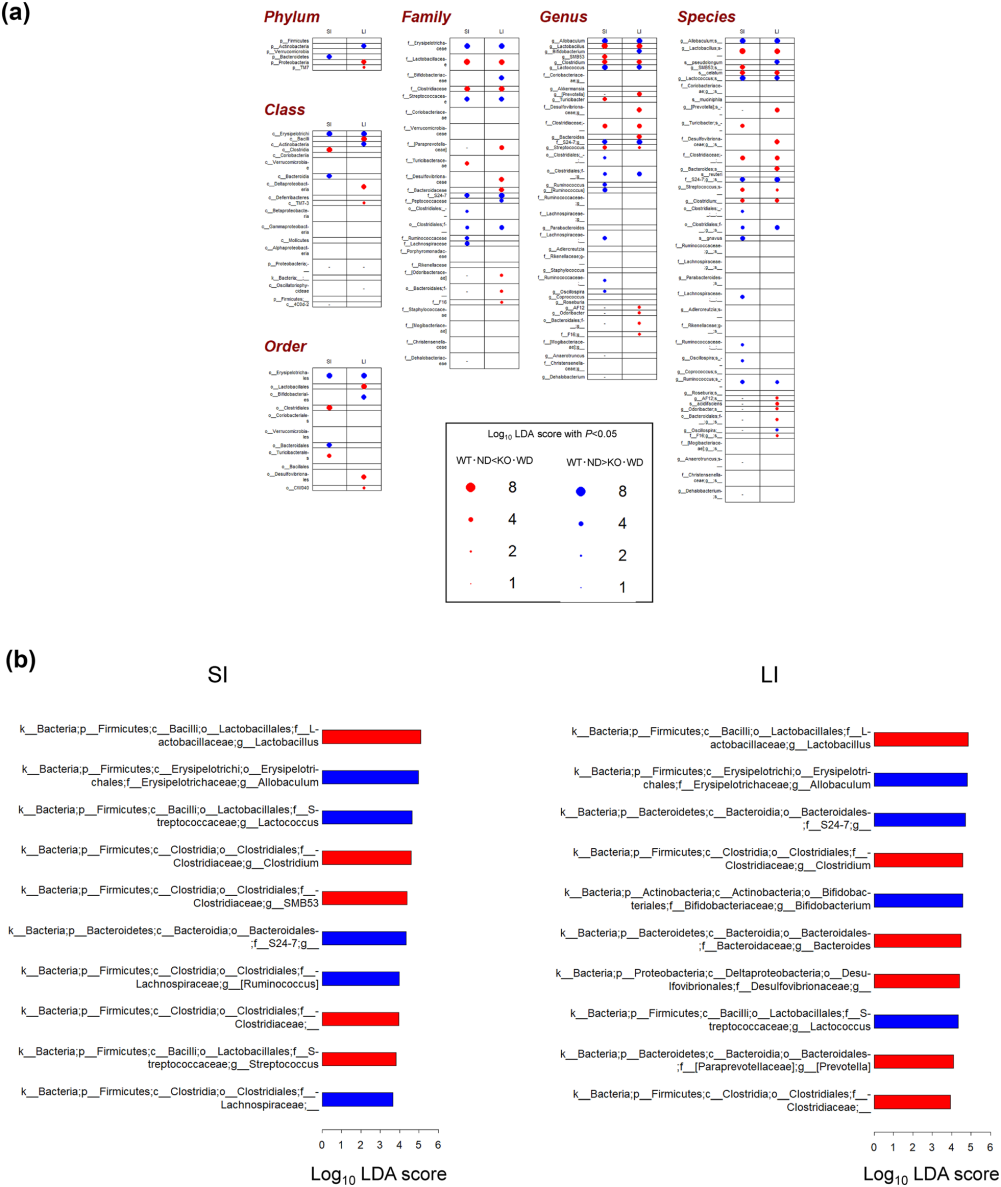
Microbial profiles in WT-ND and KO-WD mice. (**A**) Taxonomic LEfSe analyses comparing gut microbiota composition between WT-ND and KO-WD mice in SI and LI contents. Each dot represents a taxon with a statistically significant difference in LDA score between groups. Blue and red dots indicate enrichment in WT-ND (n = 6) and KO-WD (n = 8) mice, respectively. (**B**) Top 10 bacteria with the highest LDA scores (p < 0.05). Among the genera shown in panel A, the top 10 taxa most differentially abundant between WT-ND and KO-WD mice are presented. Panel A shows bacteria in the SI, and panel B shows those in the LI. Each bar represents a taxon with its corresponding LDA score on the x-axis. Red and blue bars indicate increased and decreased abundance in KO-WD and WT-ND mice, respectively. The taxonomic annotation includes kingdom (**k**), phylum (**p**), class (**c**), order (**o**), family (**f**), and genus (**g**), with the lowest-level taxon presented at the end

Using the feature table obtained from 16S rRNA gene sequencing (23,000–35,000 features per sample), microbiome analysis revealed significant compositional changes in the gut bacterial communities of WT-ND mice compared to those of the other three groups (Fig. 4 and 5), suggesting that dietary factors and MC4R deficiency contribute to distinct gut microbiota profiles that may influence host metabolism and inflammation, similar to the findings for the metabolites. When alpha diversity was compared between WT-ND mice and the other three groups, no significant differences were observed in the Shannon index or Pielou’s evenness. In contrast, richness was significantly higher in WT-ND mice than in KO-WD mice only in the small intestinal contents (SI) (FDR-adjusted p < 0.05). Beta-diversity analyses based on Jaccard distance, Bray–Curtis distance, unweighted UniFrac distance, and weighted UniFrac distance all showed significant differences between WT-ND mice and the other three groups (FDR-adjusted p < 0.05), as reflected in the principal coordinates analysis (PCoA) shown in Fig. 4. At the phylum level, LEfSe analysis revealed a marked decrease in the relative abundance of Bacteroidetes in the SI and Actinobacteria in the large intestinal contents (LI) of KO-WD mice. In contrast, the relative abundance of Proteobacteria was significantly elevated in the LI of KO-WD mice, a shift often associated with dysbiosis and systemic inflammation. Additionally, Saccharibacteria levels increased in the LI group, further underscoring the dysbiotic nature of the gut microbiome in these mice. These shifts are indicative of an altered microbial composition that could promote inflammatory and metabolic imbalance, potentially exacerbating the liver pathology associated with MASH. In particular, an increase in Proteobacteria has been linked to both acute and chronic inflammatory conditions in the gut and liver [16]. At the class level, the abundance of *Erysipelotrichic bacilli* (*Erysipelotrichia*) was reduced in both the SI and LI of KO-WD mice, which is noteworthy because members of this class have been implicated in metabolic health and inflammation [17]. Similarly, the order Erysipelotrichales and several families, including Erysipelotrichaceae, Streptococcaceae, Muribaculaceae, and Clostridiales, showed significant decreases in both SI and LI of KO-WD mice. In particular, Erysipelotrichaceae abundance was decreased in both SI and LI of KO-WD mice. Members of this family have been linked to altered lipid metabolism and metabolic inflammation; however, their role in metabolic health remains controversial, with studies reporting both beneficial and detrimental associations depending on the experimental context [18–20]. Conversely, Lactobacillaceae and Clostridiaceae were significantly increased in both the SI and LI of the KO-WD mice. These families are known for their roles in the fermentation of dietary fiber and the production of SCFAs, which are beneficial in maintaining intestinal health [21]. The increase in these families may represent a compensatory mechanism in response to dietary shifts and gut microbial alterations in KO-WD mice. At the genus level, a significant reduction in the abundance of *Allobaculum* and members of the Muribaculaceae family was observed in both the SI and LI of KO-WD mice. These genera are traditionally associated with beneficial effects on gut health, such as the production of SCFAs and the regulation of intestinal permeability [6]. This decrease in abundance may have contributed to the observed dysbiosis and impaired metabolic homeostasis in the KO-WD group. In contrast, *Lactobacillus* and *Clostridium* species were notably increased in the SI of KO-WD mice. *Lactobacillus* is a well-known probiotic genus that plays a key role in maintaining the intestinal barrier and modulating immune responses [21]. *Clostridium*, which includes both beneficial and pathogenic species, has been implicated in various aspects of gut metabolism and inflammation. The increase in the abundance of these genera may reflect an imbalance that exacerbates the inflammatory environment within the gut and liver [6]. Similar patterns were observed in the LI with additional enrichment of *Bacteroides* and Desulfovibrionaceae in KO-WD mice. *Bacteroides* are often associated with the degradation of complex polysaccharides and fibers and their increased abundance in the LI of KO-WD mice may be linked to altered carbohydrate metabolism and inflammation [22]. Desulfovibrionaceae, in contrast, are sulfate-reducing bacteria that produce hydrogen sulfide, a metabolite known to contribute to gut inflammation and intestinal barrier dysfunction [23]. Enrichment of these genera further supports the hypothesis that the gut microbiome is significantly altered in KO-WD mice, potentially contributing to the development and progression of MASH. The microbiome shifts observed in the KO-WD group were consistent with previous findings in both rodent models and human MASH, where gut microbial dysbiosis has been implicated in the development of liver diseases and metabolic disorders [24]. The top ten genera showing the greatest changes are shown in Fig. 5B. Notably, the increase in *Lactobacillus* and decrease in *Allobaculum* described above were the most prominent alterations observed in both the SI and LI. Dysbiosis has been shown to disrupt the gut–liver axis, leading to altered immune responses, increased intestinal permeability, and translocation of bacterial products such as LPS to the liver, promoting inflammation and liver damage [4]. Moreover, specific microbial genera such as *Lactobacillus*, *Clostridium*, and *Bacteroides* have been implicated in lipid metabolism, inflammation, and modulation of host immune responses [25]. These findings suggest that the microbial shifts observed in KO-WD mice play a crucial role in modulating lipid metabolism and inflammatory signaling pathways, which are central to the pathogenesis of MASH. Further studies are required to explore the causal relationships between these microbiome changes and liver pathology and to determine whether modulation of the gut microbiome can provide therapeutic benefits for MASH. In line with this interpretation, we further assessed endotoxin-related signaling. Although portal blood samples were not available and liver tissues for TLR4 expression analysis were lost, circulating plasma LPS levels were measured after 6 weeks of dietary intervention. Plasma LPS concentrations were higher in WD-fed groups than in ND-fed groups (Table 1). Beyond individual lipid alterations, our integrative metabolomic profiling revealed that multiple metabolic pathways were concurrently altered in both intestinal and hepatic compartments, suggesting shared gut–liver metabolic signatures. In particular, pathways related to bile acid metabolism, acylcarnitine accumulation, and tryptophan metabolism were prominently affected in WD-fed MC4R KO mice. These pathways are closely associated with intestinal barrier integrity, hepatic inflammation, and metabolic homeostasis through bile acid receptor (FXR/TGR5) and aryl hydrocarbon receptor (AhR) signaling [26]. Such cross-compartment metabolic reprogramming has not been previously described in MC4R-deficient MASH models and may represent novel biomarkers reflecting gut–liver axis dysfunction during disease progression. These findings provide additional mechanistic insights beyond conventional lipid alterations and underscore the importance of gut–liver metabolic communication in MASH pathogenesis.

### Functional prediction of microbial metabolic potential

**Fig. 6:**
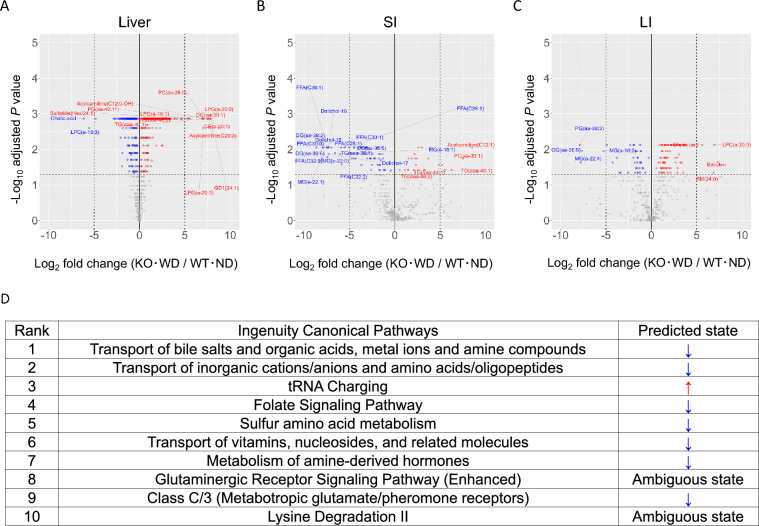
Metabolomic alterations in liver, SI, and LI and pathway enrichment analysis for the liver. (**A–C**) Volcano plots depicting differentially abundant metabolites between KO-WD (n = 8) and WT-ND (n = 6) mice in the (**A**) liver, (**B**) SI, and (**C**) LI. Each dot represents a metabolite, the x-axis indicates a log_2_ fold change (KO-WD vs. WT-ND), and the y-axis shows-log 10 of the Benjamini–Hochberg-adjusted P value. Statistical comparisons are performed using Wilcoxon rank-sum tests. Red and blue dots represent significantly increased and decreased metabolite levels, respectively (adjusted P < 0.05). The names of metabolites with |log_2_ fold change|≥ 5 are shown. The complete list of detected metabolites (including those not reaching statistical significance) is provided in Tables S1–S3. (**D**) Canonical pathway enrichment analysis using Ingenuity Pathway Analysis was conducted based on significantly altered liver metabolites. The top-ranked pathways are shown along with their predicted activation states. The blue and red arrows indicate predicted inhibition and activation, respectively, based on the z-score algorithm. The analysis revealed coordinated downregulation of key hepatic pathways related to amino acid metabolism and lipid processing in KO-WD mice, with only tRNA charging predicted to be activated

To explore the functional consequences of microbiome shifts, we summarized the metabolomic alterations in the liver, SI, and LI of KO-WD and WT-ND mice, and performed pathway enrichment analysis for the liver (Fig. 6). These analyses allowed us to assess the regional differences in microbial metabolic output and their potential impact on host metabolism and immune responses. The alterations of each metabolite are described in the section "Tissue-Specific Metabolomic Alterations.”

**Fig. 7:**
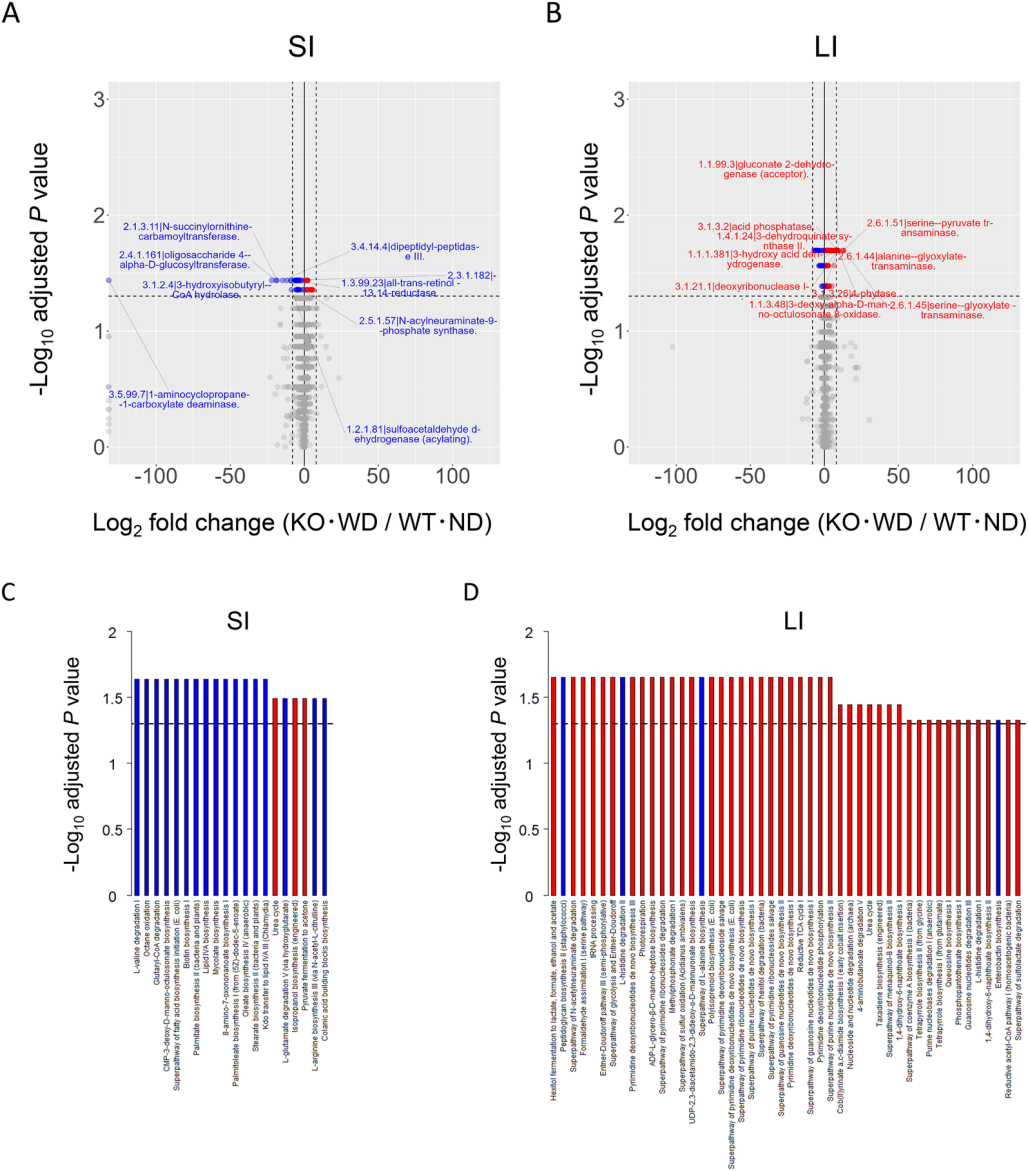
Volcano plots of bacterial enzyme profiles predicted by PICRUSt2. Differentially enriched enzyme-coding genes (**A-B**) or pathways (**C-D**) between WT-ND and KO-WD mice were inferred using the Phylogenetic Investigation of Communities by Reconstruction of Unobserved States 2 (PICRUSt2) based on 16S rRNA gene sequencing data of intestinal contents. A and C show data from SI and B and D show data from LI. The x-axis represents the log₂ fold change (KO-WD vs. WT-ND) and the y-axis shows the-log10 of the adjusted P-value. Enzyme features are assigned using EC (Enzyme Commission) numbers, and statistical comparisons are performed using the Wilcoxon rank-sum test with Benjamini–Hochberg correction for multiple testing. The dashed horizontal lines in C and D indicate an adjusted P-value of 0.05. The complete list of predicted enzyme features (including those not reaching statistical significance) is provided in Tables S4 and S5

Enzyme prediction using PICRUSt2 further revealed that bacterial enzymes involved in amino acid and carbohydrate metabolism were differentially abundant between the two groups (Fig. 7A and B). It should be noted that these predictions are based on inference and do not represent direct measurements of enzymatic activity, but rather provide a putative functional profile. Several enzymes that were estimated to show large changes in the SI, including 1-aminocyclopropane-1-carboxylate deaminase (EC:3.5.99.7), 3-hydroxyisobutyryl-CoA hydrolase (EC:3.1.2.4), and oligosaccharide 4-alpha-D-glucosyltransferase (EC:2.4.1.161), were significantly reduced in KO-WD mice compared to that in WT-ND mice. These enzymes are involved in critical metabolic processes such as amino acid degradation and carbohydrate utilization, which are important for maintaining gut health and energy homeostasis. The reduction in these enzyme levels suggests a disruption in the normal metabolic pathways in the small intestine of KO-WD mice, potentially impairing nutrients and energy balance [27]. In contrast, several enzymes that were estimated to show large changes in the LI of KO-WD mice showed increased levels of enzymes, including alanine-glyoxylate transaminase (EC:2.6.1.44), serine-glyoxylate transaminase (EC:2.6.1.45), and serine-pyruvate transaminase (EC:2.6.1.51) compared to WT-ND mice (Figs. 7A and B). These enzymes are involved in amino acid metabolism and in the interconversion of key metabolic intermediates such as pyruvate and glyoxylate. The increased levels of these enzymes in the LI suggest a shift towards altered amino acid metabolism, which may be associated with the inflammatory responses observed in KO-WD mice. Furthermore, differential enzyme expression in the SI and LI suggests region-specific reprogramming of microbial metabolic output in response to dietary and genetic factors present in KO-WD mice. This regional specificity is consistent with previous studies that have shown how the gut microbiota can influence metabolic and immune responses in a localized manner. These differences were also suggested by pathway-level analyses (Figs. 7C and D). Pathway enrichment indicated that pathways involved in amino-acid metabolism and carbohydrate metabolism differed between the SI and LI. We also observed differences in pathways related to SCFA metabolism and LPS biosynthesis. Notably, many of these pathways tended to be decreased in the SI but increased in the LI. This reprogramming of microbial metabolic pathways may have profound implications for host metabolism and immune functions, particularly in the context of liver diseases [28]. Comparison with human MASH cohorts further supports the translational relevance of our findings. Clinical studies have consistently reported enrichment of Enterobacteriaceae and Desulfovibrionaceae (Gram-negative taxa capable of LPS production) and depletion of SCFA–producing genera such as *Faecalibacterium* in patients with MASH. Additionally, dysregulation of bile-acid and tryptophan metabolism has been observed in both fecal and plasma metabolomes of human subjects, correlating with hepatic inflammation and fibrosis severity [6, 29–34]. The WD-fed MC4R KO mice exhibited similar compositional and metabolic alterations, including enrichment of Desulfovibrionaceae and perturbation of bile-acid–related pathways, suggesting that this model faithfully recapitulates key microbiome–metabolome features of human MASH. These parallels emphasize the translational potential of the MC4R KO model as a preclinical platform for investigating microbiota-driven mechanisms and for evaluating therapeutic interventions targeting the gut–liver axis. For example, microbial enzymes involved in carbohydrate fermentation and SCFA production were more abundant in SI, whereas those related to amino acid metabolism were enriched in LI. This partitioning of metabolic functions across different gut regions likely contributes to the diverse physiological roles of the gut microbiome in maintaining metabolic health [35]. The shifts in predicted microbial enzyme abundances observed in KO-WD mice are particularly relevant in the context of metabolic diseases, such as MASH. The increased abundance of enzymes involved in amino acid metabolism in the LI might promote the production of metabolites that influence liver inflammation and steatosis. This suggests that microbial metabolic shifts affect local gastrointestinal processes and may have systemic consequences on liver health. Recent studies have highlighted the role of microbially derived metabolites, such as SCFAs and amino acids, in modulating host immune responses and liver inflammation [36]. Moreover, bacterial enzymes involved in the metabolism of specific amino acids and carbohydrates have been implicated in the regulation of host energy balance and immune response [37]. For example, certain bacterial species can synthesize metabolites that influence the activity of immune cells, thereby contributing to the regulation of inflammatory pathways in the liver and other tissues. Alterations in microbial metabolism observed in KO-WD mice may therefore provide insights into the mechanistic links between the gut microbiome and liver disease progression, suggesting potential therapeutic avenues for managing MASH.

### Network analysis of gut–liver metabolic axis

**Fig. 8:**
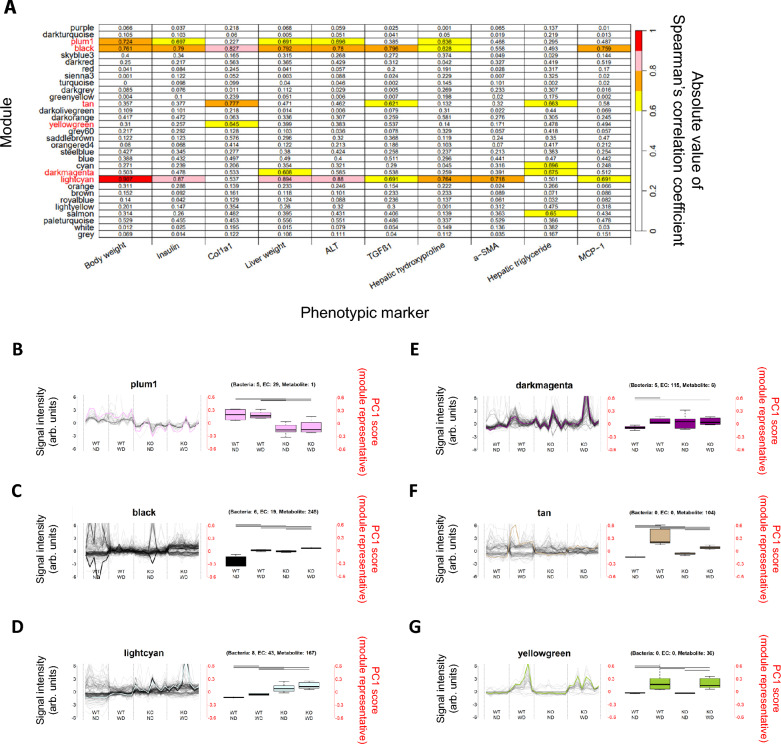
Correlation between WGCNA modules and phenotypic markers, and perturbation profiles of representative modules. (**A**) Heatmap showing the absolute values of Spearman’s correlation coefficients between the PC1 score, representing each of the 31 WGCNA modules (rows) and host phenotypic markers (columns). Color intensity represents the magnitude of positive correlation, with values approaching 1 (red). Several modules exhibited moderate to strong correlations with hepatic fibrosis markers, lipid accumulation, or inflammation. (**B–G**) Signal intensity profiles and compositional features of selected modules that demonstrated correlations in panel A and showed significant differences between KO-WD and WT-ND. For each module, the left plot shows the PC1 score as the representative module stratified by experimental group: WT-ND (n = 6), WT-WD (n = 6), KO-ND (n = 8), and KO-WD (n = 8). The thick colored lines denote the module PC1 score, whereas the grey lines represent the robust z-scores of individual molecular or bacterial features. The boxplot (right) summarizes the PC1 score profiles for each experimental group. Significant differences between groups are indicated by the lines above the boxplots. The number of contributing features within each module, including taxonomic (bacteria), enzymatic (**EC**), and (metabolite) elements, is shown at the top of each boxplot. (**B**) plum1: shows a prominent alteration in the KO groups (KO-ND and KO-WD) compared to the WT groups (WT-ND and WT-WD), with contributions from 5 bacterial taxa, 29 enzyme orthologs, and 1 metabolite. This module represents molecular and bacterial groups affected by MC4R KO. (**C**) Black and (**D**) light cyan: markedly altered compared to WT-ND, especially in KO-WD, suggesting that these modules are highly metabolite-enriched and affected by both MC4R KO and diet. The black module comprises 6 bacterial taxa, 19 ECs, and 245 metabolites, whereas the light cyan module comprises 8 bacterial taxa, 43 ECs, and 167 metabolites. (**E**) Dark magenta: moderately altered from WT-ND; composed of 5 bacterial taxa, 115 ECs, and 6 metabolites. It is affected by both MC4R KO and diet, although to a lesser extent. (**F**) Tan and (**G**) yellow-green: Both modules are almost exclusively composed of metabolite features (104 and 36 metabolites, respectively) with no bacterial or enzymatic contributions. These modules represent molecular groups primarily affected by diet

**Fig. 9:**
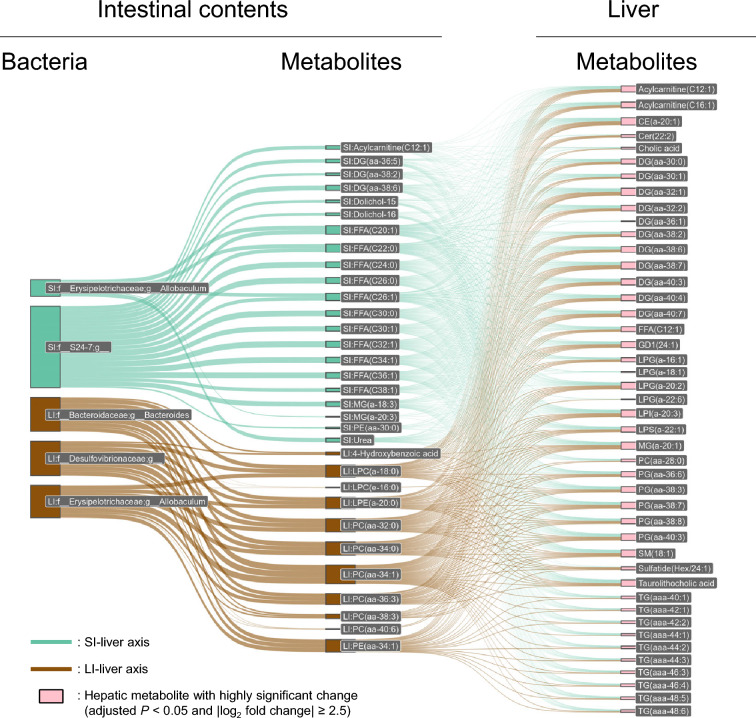
Gut–liver axis linked to markedly perturbed hepatic metabolites and liver phenotypes. Modules identified by WGCNA that were significantly correlated with hepatic fibrosis, inflammation, or lipid accumulation (Fig. 8A) were further examined to extract representative bacterial and metabolite features from the small and large intestinal contents. Shown are bacterial taxa and metabolites from intestinal contents (left and middle columns) and corresponding liver metabolites (right column) that exhibited highly significant changes and strong associations (|Spearman’s correlation coefficient|≥ 0.6) within each gut–liver axis. Not all molecules along the gut–liver axis are shown; bacterial enzymes and some metabolites present in the gut and liver are omitted. Upper panel: SI–liver axis Bacteria and metabolites within this axis in the SI were deficient in KO-WD mice and included representative bacteria such as *Allobaculum* (family Erysipelotrichaceae, phylum Firmicutes) and members of the Muribaculaceae group (phylum Bacteroidetes). Metabolites in SI include multiple species of free fatty acids, acylcarnitine (e.g., C12:1), urea, dolichols, and phosphatidylethanolamine (PE(aa-30:0)). These were highly correlated with altered hepatic metabolites, including taurolithocholic acid, cholic acid, acylcarnitines, LPGs, PGs, and multiple triglycerides (TGs). Lower panel: LI–liver axis The bacteria and metabolites within this axis in the LI included representative large intestinal bacterial taxa from Bacteroidaceae (enriched in KO-WD mice), *Allobaculum* (deficient in KO-WD mice), and Desulfovibrionaceae (phylum Proteobacteria, enriched in KO-WD mice). Metabolites in the large intestine included 4-hydroxybenzoic acid (deficient in KO-WD mice) and lysophospholipids such as LPCs, LPEs, PCs, and PEs (enriched in KO-WD mice). These features are associated with hepatic lipid classes, including phosphatidylglycerols, PCs, PEs, DGs, TGs, and sulfated hexoses, many of which are perturbed in response to Western diet in MC4R-deficient mice. These data indicate compartment-specific associations between intestinal microbial and metabolic environments and hepatic metabolic disruption, supporting the concept that distinct SI and LI liver axes contribute to MASH pathogenesis in this model

**Fig. 10:**
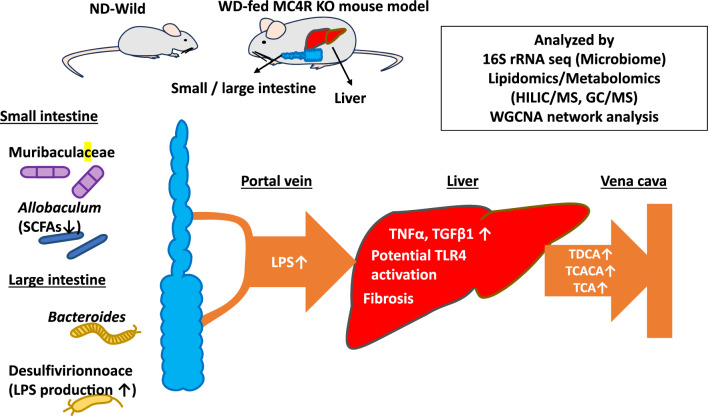
Intestinal dysbiosis and metabolomic alterations contributing to hepatic steatosis and fibrosis in WD-fed MC4R-deficient mice. Dysbiosis in the gut microbiota and associated metabolite shifts were observed in both the SI and LI of WD-fed MC4R KO mice. In the small intestine, reductions in fatty acyl metabolites and the bacterial Muribaculaceae family are prominent, corresponding to modules associated with hepatic inflammation and lipid accumulation. In contrast, the large intestine exhibited increased glycerophospholipid levels and enrichment of *Bacteroides* and Desulfovibrionaceae, along with a decrease in *Allobaculum*, which were associated with hepatic fibrosis-related metabolomic changes. These spatially distinct perturbations along the gut–liver axis underscore the compartmentalized contribution of the intestinal environment to MASH pathogenesis in this model

To understand the interplay between gut-derived signals and hepatic metabolic disturbances, we performed WGCNA of the microbiota, predicted microbial enzymes, and metabolites across the intestinal contents and hepatic and plasma compartments. WGCNA is a powerful tool for identifying modules of co-expressed genes or metabolites that exhibit similar patterns of variation and can provide insights into the functional relationships between microbial communities and host metabolic processes [38]. A total of 31 modules were identified based on these covariation patterns, which were further analyzed for their correlations with various phenotypes associated with MASH, such as ALT, insulin levels, and hepatic triglyceride and hydroxyproline contents (Fig. 8). Six of these modules (plum1, black, tan, yellow-green, dark magenta, and light cyan) showed correlations with MASH-associated phenotypes, as summarized in Table 6. Two additional modules among the remaining modules (cyan and salmon) also showed correlations with hepatic triglycerides, which are one of the MASH-associated phenotypes. However, the molecules and bacteria belonging to these two modules did not show significant differences between KO-WD and WT-ND; therefore, we did not focus on these modules in subsequent analyses. These results highlight the complex and multifaceted relationship between the gut microbiota, metabolites, and liver disease progression. Next, we focused on the bacteria, enzymes, and metabolites belonging to the six modules that were found to be associated with relevant phenotypes and constructed a network representing the gut–liver axis. Specifically, we comprehensively extracted networks connecting intestinal metabolites, enzymes, and bacteria that correlated with metabolites exhibiting marked changes in the liver (adjusted *P*-value < 0.05 and |log_2_ fold change|≥ 2.5) (Fig. 9). Notably, a correlation was observed between the small intestinal abundances of *Allobaculum* and Muribaculaceae and several intestinal metabolites, including free fatty acids (FFAs), diacylglycerols (DGs), and monoglycerides (MGs). The abundance of these specific bacterial genera positively correlated with the levels of intestinal FFAs, DGs, and MGs, which are important intermediates in lipid metabolism [39]. Furthermore, these intestinal metabolites correlated with hepatic lipid species, such as lysophosphatidylglycerols (LPG), phosphatidylglycerols (PG), DG, and triglycerides (TG) (Fig. 9). This suggests that the microbial communities in the small intestine directly influence the hepatic lipidome, potentially contributing to the onset and progression of hepatic steatosis and inflammation. These findings are consistent with those of previous studies, indicating that the gut microbiota plays a key role in regulating lipid metabolism through the production of microbial metabolites, which in turn affect host metabolic processes [40]. In the large intestine, increased abundances of *Bacteroides* and Desulfovibrionaceae were observed, which were associated with altered levels of liver lysophosphatidylcholines (LPCs) and phosphatidylcholines (PCs), which are critical components in the development of liver inflammation and steatosis [41]. The correlation between these bacterial genera and hepatic lipid species further supports the concept that gut microbiota can directly influence hepatic lipid metabolism and contribute to the pathogenesis of liver diseases, particularly NAFLD and MASH [42]. These findings underscore the pivotal roles of specific microbial communities and their associated metabolic networks in modulating hepatic function and driving disease progression (Fig. 10). By influencing the gut–liver axis, these microbial-metabolic interactions may directly shape the hepatic lipidome and play a central role in orchestrating the progression of liver diseases, such as MASH. The identification of these networks provides new opportunities for developing therapeutic strategies targeting the gut microbiota to manage metabolic liver diseases. Because the networks identified in this study are correlational, definitive causal relationships must be established through perturbation studies that manipulate the gut microbiota and candidate metabolites, for example, antibiotic-mediated microbiota depletion and reconstitution, fecal microbiota transplantation (FMT), and targeted supplementation or depletion of specific bile acids and lipid intermediates. Ideally, such experiments should be implemented in time-resolved designs and combined with isotopic tracing, which would clarify whether the modules identified here act as causal drivers of steatosis, inflammation, and fibrosis, or instead represent secondary consequences of disease progression. Indeed, recent studies have demonstrated that interventions aimed at modulating gut microbiota composition can mitigate liver inflammation and fibrosis, suggesting that microbiome-targeted therapies may be a promising approach for treating MASH [43]. Overall, this network analysis provides valuable insights into the complex interactions between the gut microbiota and liver, highlighting potential microbial and metabolic targets for therapeutic intervention in MASH and other metabolic liver diseases. These results also suggest that microbial-driven metabolic disturbances in the gut can serve as early indicators of liver disease progression and provide opportunities for early detection and intervention [44].

**Table 6:** Correlation between each module classified by WGCNA and phenotypic markers

Module	Body weight	Plasma insulin	Col1a1 mRNA	Liver weight	ALT	TGFβ1 mRNA	HP	α-SMA mRNA	TG	MCP-1 mRNA
plum1	**0.72**	**0.70**	0.23	**0.69**	**0.70**	0.39	**0.64**	0.47	0.30	0.49
black	**0.76**	**0.79**	**0.83**	**0.79**	**0.78**	**0.80**	**0.63**	0.56	0.49	**0.76**
tan	0.36	0.38	**0.78**	0.47	0.46	**0.62**	0.13	0.32	**0.66**	0.58
yellow green	0.31	0.26	**0.64**	0.40	0.38	0.54	0.14	0.17	0.48	0.49
dark magenta	0.50	0.48	0.53	**0.61**	0.58	0.54	0.26	0.39	**0.68**	0.51
light cyan	**0.91**	**0.87**	0.54	**0.89**	**0.88**	**0.69**	**0.76**	**0.72**	0.50	**0.69**

Summary of the correlations between the representative values (first principal component scores) of each module classified by WGCNA and the phenotypic markers measured in this study. Only modules that showed correlations with phenotypic markers and significant differences between the KO-WD and WT-ND groups were included. Correlations are represented as absolute values of Spearman’s correlation coefficients. Values with absolute Spearman correlation coefficients of 0.6 or greater are indicated in bold and underlined. HP: Hydroxy proline (hepatic), TG: Triglyceride (hepatic)

## Discussion

In this study, we conducted a comprehensive microbiome and metabolome analysis of WD-fed MC4R KO mice. This murine model closely recapitulates the histopathological and metabolic characteristics of human MASH, including obesity, insulin resistance, hepatic steatosis, inflammation, and fibrosis [9]. The relevance of the model to human MASH pathology has been validated in previous reports, including a recent comprehensive evaluation [8], which ranked it among the most pathophysiologically representative preclinical models. By analyzing intestinal and hepatic samples across two (WT-ND and KO-WD) or four experimental groups (WT-ND, WT-WD, KO-ND, and KO-WD), our integrated approach allowed us to investigate the dynamic interactions between gut microbiota composition, bile acid metabolism, lipid absorption, as well as liver pathology. The MC4R-deficient mouse model fed a Western diet has been proposed as a representative preclinical model of advanced MASH; however, its translational relevance requires careful contextualization against previously reported disease severity metrics. In this regard, prior studies have demonstrated that WD- or HFD-fed MC4R KO mice develop histopathological features consistent with advanced steatohepatitis. Matsumoto et al. reported that WD-fed MC4R KO mice exhibited a NAFLD activity score (NAS) of approximately 4.0, whereas Yamada et al. demonstrated that MC4R KO mice fed a high-fat diet reached an NAS of 5, closely reflecting the degree of hepatic steatosis, inflammation, and ballooning observed in patients with MASH [45, 46].

Consistent with these reports, our KO-WD mice displayed a comprehensive phenotype encompassing obesity, insulin resistance, elevated liver injury markers, fibrogenic activation, and marked upregulation of fibrosis- and inflammation-associated genes. Notably, this integrated disease profile was accompanied by increased circulating LPS levels, suggesting enhanced metabolic endotoxemia, a key pathogenic driver linking gut dysbiosis to hepatic inflammation in human MASH. The convergence of metabolic, inflammatory, and fibrotic features observed in this study supports the translational relevance of the WD-fed MC4R KO model as a robust platform for investigating gut–liver axis dysfunction and for evaluating therapeutic interventions targeting advanced stages of MASH. These analyses uncovered a constellation of microbiota and metabolite signatures that distinguished MASH-like phenotypes and yielded potential mechanistic insights into gut–liver axis dysregulation in this disease context. Our findings align with the increasing recognition of the gut–liver axis as a pivotal player in the pathogenesis of metabolic diseases. Dysbiosis of the intestinal microbiota contributes to insulin resistance and liver inflammation and affects bile acid metabolism, influencing lipid homeostasis and liver fibrosis. Here, we provide evidence that gut microbiota plays a critical role in modulating MASH progression in an MC4R KO model. Notably, alterations in microbiota composition, lipid metabolism, and bile acid profiles were strongly associated with phenotypic changes in the liver, suggesting a multifaceted role for gut microbes in exacerbating the disease. Notably, PICRUSt2-based functional profiling reflects inferred functional potential derived from 16S rRNA data and does not provide direct evidence of gene expression or enzymatic activity; therefore, these results should be interpreted as predictions rather than as measured biological activity. Accordingly, we discuss PICRUSt2-predicted functions only as hypothesis-generating estimates and we interpret them in conjunction with metabolomic measurements and relevant literature, avoiding activity-based or causal conclusions. Regarding the microbiota signatures and gut dysbiosis in MASH, our findings revealed significant alterations in the intestinal microbial composition of WD-fed MC4R KO mice, including a consistent reduction in Muribaculaceae and an increase in *Lactobacillus* in both the small and large intestines. Some bacteria showed changes only in one of the intestinal contents, including *Bacteroides* and Desulfovibrionaceae, which showed a significant increase specifically in the large intestine. Among these alterations, the change in Muribaculaceae levels resembles the microbiota shifts observed in human obesity and type 2 diabetes, in which reduced Muribaculaceae abundance is inversely correlated with BMI, waist circumference, and insulin resistance [47]. The significance of these microbial changes is underscored by their correlations with hepatic pathology in our model; Muribaculaceae and *Allobaculum* abundances were negatively associated with hepatic steatosis, inflammation, and fibrosis, whereas *Bacteroides* and Desulfovibrionaceae exhibited strong positive correlations. These bacteria are known to produce metabolites that directly affect gut barrier integrity and hepatic inflammation. For example, Desulfovibrionaceae may produce endotoxins, such as LPS, which may trigger Toll-like receptor 4 (TLR4)-mediated inflammatory responses in hepatic Kupffer cells and hepatocytes, contributing to MASH progression [48]. Moreover, *Bacteroides* are implicated in bile acid deconjugation and fermentation of polysaccharides, leading to the production of SCFAs, which may have context-dependent effects on metabolic homeostasis [49]. In addition to the changes observed in Muribaculaceae and *Lactobacillus*, Fig. 5 demonstrates pronounced alterations in Clostridial taxa across both the small and large intestinal compartments of WD-fed MC4R KO mice. Members of the order *Clostridiales*, including families such as *Clostridiaceae*, were significantly enriched in KO-WD mice in both SI and LI, indicating a broad restructuring of anaerobic bacterial communities along the intestinal tract.

Clostridia are functionally diverse and play pivotal roles in intestinal homeostasis through SCFA production, secondary bile acid transformation, and maintenance of gut barrier integrity. Certain Clostridial species are major producers of butyrate, a key energy source for colonocytes with anti-inflammatory properties; however, other members possess bile acid–transforming enzymes, including 7α-dehydroxylases, which generate secondary bile acids capable of modulating FXR and TGR5 signaling. Dysregulated expansion of specific Clostridial taxa may therefore contribute to altered bile acid pools and impaired gut barrier function, facilitating increased intestinal permeability and translocation of microbial products such as LPS.

Consistent with this notion, the enrichment of Clostridial taxa in KO-WD mice occurred in parallel with elevated circulating LPS levels and marked hepatic inflammation, supporting a functional link between Clostridia-driven metabolic outputs and gut–liver axis dysregulation. Together, these findings suggest that shifts in Clostridial communities represent a key microbiome feature associated with metabolic endotoxemia, bile acid dysregulation, and MASH progression in the WD-fed MC4R KO model.

Notably, although altered Firmicutes/Bacteroidetes (F/B) ratios are frequently cited in the literature as a hallmark of obesity and NAFLD, Firmicutes levels were not significantly changed, whereas Bacteroidetes levels were decreased. These findings suggest that the F/B paradigm does not fully capture the complexity of microbial shifts in genetically obese diet-induced steatohepatitis models. Compared to the methionine-choline-deficient (MCD) model, which induces hepatic steatosis and inflammation in the absence of obesity or insulin resistance, the KO-WD model more faithfully replicates the microbiome profile of metabolically unhealthy obesity. Reductions in Muribaculaceae and *Lactobacillus* have been documented in MCD-fed mice [50], whereas our KO-WD mice showed elevated *Lactobacillus* levels, which is consistent with the human metabolic syndrome. These findings highlight the importance of model selection in microbiome studies and support the translational value of the MC4R KO model. In terms of lipid metabolism and altered intestinal absorption, liver metabolomics revealed marked increases in TGs, DGs, and glycerophospholipids, specifically phosphatidylglycerol, and lysophosphatidylglycerol, in KO-WD mice. These hepatic lipid alterations were accompanied by reduced levels of intestinal FFAs and MGs, suggesting enhanced intestinal absorption and possibly increased hepatic esterification, indicating a shift toward greater lipid uptake and storage. The accumulation of saturated TG species observed in our model is consistent with the patterns observed in the liver biopsies of patients with MASH [51], where increased hepatic de novo lipogenesis and impaired lipid export have been proposed as contributing mechanisms. Additionally, phospholipid remodeling in the liver, particularly increased PG levels, may reflect altered membrane composition and endoplasmic reticulum stress, which are increasingly recognized as key factors in MASH pathogenesis [52]. However, studies by Vvedenskaya et al. [53] and Gorden et al. [54] have suggested that phospholipid changes in human MASH are modest, implying that some of the lipidomic signatures in our model may be species- or diet-specific. Nonetheless, the directionality of the changes, increased hepatic TGs and decreased intestinal FFAs, parallels the human data, reinforcing the relevance of the model. For disruption of bile acid homeostasis and hepatic transporters, in line with these lipidomic findings, we observed a substantial elevation in plasma levels of taurine-conjugated bile acids—including taurocholic acid, taurodeoxycholic acid, and taurochenodeoxycholic acid—in KO-WD mice. Although mice predominantly synthesize taurine-conjugated bile acids, whereas humans favor glycine conjugation, this elevation mirrors the accumulation of conjugated bile acids observed in patients [55]. These bile acids are known to activate the farnesoid X receptor (FXR) and Takeda G-protein coupled receptor 5 (TGR5), which regulate lipid metabolism, inflammation, and glucose homeostasis. This dysregulation may contribute to hepatic insulin resistance and fibrogenesis [56]. Plasma bile acid levels may be increased by the downregulation of hepatic NTCP (SLC10A1), which is a sodium-dependent bile acid transporter responsible for the uptake of conjugated bile acids into hepatocytes. When NTCP expression decreases, it can hinder the hepatic clearance of circulating bile acids, thereby exacerbating their systemic accumulation. This hypothesis is consistent with previous reports on animal models and human liver tissues from patients with MASH [57, 58]. Furthermore, elevated levels of FXR-regulated genes, such as small heterodimer partner and fibroblast growth factor 15 in the ileum suggest a compensatory feedback loop in bile acid signaling, potentially affecting hepatic bile acid synthesis through CYP7A1 repression [59].

On implications for therapeutic targeting of the gut–liver axis, our data underscore the intricate crosstalk between microbial populations, intestinal lipid handling, bile acid metabolism, and liver pathology. The several microbial and metabolite signatures identified in this study represent potential therapeutic targets. For instance, the modulation of bile acid pools using FXR agonists such as obeticholic acid or FGF19 analogs has been actively investigated in MASH clinical trials [60]. Similarly, microbiota-directed therapies, including the use of SCFA-producing probiotics, bile acid-converting consortia, and endotoxin-neutralizing agents, may offer novel routes for reducing hepatic inflammation and fibrosis. Elevated levels of endotoxin-producing Desulfovibrionaceae in KO-WD mice further supported the relevance of these strategies. These findings open several avenues for the development of microbiome-targeted interventions as adjunct therapies for MASH. Regarding model strengths, limitations, and future directions, in this study, we represent the first integrated gut–liver axis analysis combining microbiome and metabolome profiling in WD-fed MC4R KO mice. Our findings validate the capacity of the model to reproduce key features of human MASH and provide a platform for dissecting host-microbe-metabolite interactions.

We cannot completely exclude the influences of breeding origin, baseline differences and strain-specific effects. Regarding the difference of breeding origin and baseline differences, all mice (both wild-type and MC4R KO) were bred and maintained under identical SPF conditions and housed in the same animal room. It has been reported that gut microbiome can rapidly respond to an altered diet, highlighting the profound influence of dietary composition on its functionality [61, 62]. Furthermore, the all mice were fed with the same batch of CE-2 for 15 days, followed by ND for 12 days before the initiation of WD feeding, thereby minimizing potential differences owing to environmental or breeding sources. Regarding baseline differences, although we cannot completely exclude it, the consistent housing and dietary conditions, together with the pronounced metabolic and histopathological alterations observed after WD feeding (Table 1), strongly suggest that the major microbial and metabolic differences were disease-related rather than strain-dependent. Regarding strain-specific effects, a limitation of the present study is that food intake was not directly measured. MC4R-deficient mice are known to display hyperphagia, especially under high-fat feeding conditions [63, 64]. Therefore, increased caloric intake likely contributed, at least in part, to the greater metabolic abnormalities observed in MC4R KO mice fed a WD. This limitation should be considered when interpreting the data; however, because all groups were fed ad libitum under identical environmental conditions, the genotype-dependent differences observed are still considered to primarily reflect intrinsic metabolic alterations.

This study has some limitations. First, the model’s reliance on a single genetic knockout and a high-fat, high-sugar diet does not capture the full heterogeneity of human MASH, which involves polygenic risk factors and variable dietary exposures. Secondly, interspecies differences in bile acid conjugation, microbiota composition, and lipid transport limit the extrapolation of some findings. Third, our analysis focuses on steady-state profiles. Future longitudinal studies are needed to clarify the causal relationships among microbiota shifts, metabolic alterations, and histological progression. Future studies should also explore the temporal dynamics of gut microbial colonization and liver injury in this model and compare these patterns with those in human patient cohorts. Fourth, although the microbiome analysis was performed using 16S rRNA sequencing, accurately distinguishing closely related taxa based solely on 16S rRNA analysis is challenging. For example, taxa sharing approximately 99% sequence (e.g., *Allobaculum* and Feacalibaculum) similarity may be identified as one or the other depending on the analysis parameters or the reference database used. Therefore, as long as the analysis relies on 16S rRNA sequences, determining precisely which taxon is present is challenging. More accurate identification would require additional approaches, such as metagenomic analysis. The integration of transcriptomics, immunophenotyping, and gut barrier function assays will further refine our understanding of gut-liver communication in MASH. Finally, therapeutic intervention studies targeting bile acid pathways, microbial populations, or metabolic enzymes in this model will help to establish their translational relevance and potential efficacy.

In this study, we demonstrated that WD-fed MC4R KO mice developed a MASH-like phenotype, characterized by key features including gut dysbiosis, increased intestinal lipid absorption, hepatic lipid accumulation, and systemic bile acid dysregulation. These findings closely mirror the pathological characteristics observed in human MASH, confirming the relevance of this model for studying the underlying mechanisms of the gut–liver axis in metabolic diseases. The significant correlations observed between specific bacterial taxa, bile acids, and liver outcomes suggest that microbiota-mediated alterations in lipid metabolism and bile acid homeostasis play pivotal roles in the progression of MASH. Our results provide valuable insights into the complex interactions between the gut microbiota and the liver, offering a strong foundation for future mechanistic studies and therapeutic strategies aimed at modulating gut-derived signals to treat or prevent MASH. Further research exploring targeted interventions that address microbiota composition, bile acid metabolism, and liver inflammation could lead to novel therapeutic approaches for managing MASH.

## Conclusion

We investigated the gut-liver axis in MASH using MC4R-KO mice fed a WD. WD-fed MC4R-KO mice developed hepatic steatosis, inflammation, and fibrosis. Specific gut microbiota changes and altered bile acid and lipid metabolism correlated with MASH severity. These findings suggest gut-liver axis dysregulation contributes to MASH progression and may represent a therapeutic target.

## Supplementary Information

Additional file 1.

## Data Availability

The metabolomics datasets supporting the conclusions of this article are available in the Metabolomics Workbench repository under datatrack_id:6965 and study_id: ST004599. The microbiome datasets supporting the conclusions of this article are available in the NCBI Gene Expression Omnibus (GEO) repository under accession number GSE316452. Additional supporting information is included within the article and its additional files.

## Abbreviations

MC4R: Melanocortin 4 receptor
KO: Knockout
WD: Western diet
MASH: Metabolic dysfunction-associated steatohepatitis
SCFA: Short-chain fatty acids
NTCP: Sodium-dependent taurocholate cotransporting polypeptide
FXR: Farnesoid X receptor
TGR5: Takeda G-protein coupled receptor 5
BAs: Bile acids
LPS: Lipopolysaccharide
TLR4: Toll-like receptor 4
TGs: Triglycerides
DGs: Diglycerides
PG: Phosphatidylglycerol
LPG: Lysophosphatidylglycerol
PE: Phosphatidylethanolamine
FFAs: Free fatty acids
MGs: Monoglycerides
F/B: Firmicutes/Bacteroidetes
PCA: Principal component analysis
WGCNA: Weighted gene co-expression network analysis
